# The Quantum Biology of Reactive Oxygen Species Partitioning Impacts Cellular Bioenergetics

**DOI:** 10.1038/srep38543

**Published:** 2016-12-20

**Authors:** Robert J. Usselman, Cristina Chavarriaga, Pablo R. Castello, Maria Procopio, Thorsten Ritz, Edward A. Dratz, David J. Singel, Carlos F. Martino

**Affiliations:** 1 Department of Chemistry and Biochemistry, Montana State University, Bozeman, MT 59717, USA; 2 Department of Biomedical Engineering, Florida Institute of Technology, Melbourne, FL 32901, USA; 3 Consejo Nacional de Investigaciones Científicas y Técnicas (CONICET), Universidad de Belgrano (UB), Villanueva 1324, C1426BMJ, Buenos Aires, Argentina; 4 Department of Physics and Astronomy, University of California, Irvine, CA 92697, USA.

## Abstract

Quantum biology is the study of quantum effects on biochemical mechanisms and biological function. We show that the biological production of reactive oxygen species (ROS) in live cells can be influenced by coherent electron spin dynamics, providing a new example of quantum biology in cellular regulation. ROS partitioning appears to be mediated during the activation of molecular oxygen (O_2_) by reduced flavoenzymes, forming spin-correlated radical pairs (RPs). We find that oscillating magnetic fields at Zeeman resonance alter relative yields of cellular superoxide (O_2_^•−^) and hydrogen peroxide (H_2_O_2_) ROS products, indicating coherent singlet-triplet mixing at the point of ROS formation. Furthermore, the orientation-dependence of magnetic stimulation, which leads to specific changes in ROS levels, increases either mitochondrial respiration and glycolysis rates. Our results reveal quantum effects in live cell cultures that bridge atomic and cellular levels by connecting ROS partitioning to cellular bioenergetics.

Quantum biology may be thought of as the signatures of molecular-level quantum phenomena observed in biological systems at functional, cellular, or organism levels^1^,^2^. For example, quantum effects in biological systems have been implicated in mechanisms for avian navigation^3^,^4^,^5^,^6^,^7^, olfactory sensing^8^,^9^, and photosynthesis^10^,^11^,^12^. Here, we present results that reveal a novel domain of quantum biology: the control of the biological production of reactive oxygen species (ROS) via coherent spin dynamics in a radical pair (RP) reaction. Our work builds on studies of complexes between reduced flavoenzymes and molecular oxygen^13^,^14^, within the context of quantum biology^15^ and the radical pair mechanism (RPM)^16^,^17^.

The RPM offers an attractive foundation to explain how quantum effects can influence biochemical ROS production. At the heart of the RPM lies a coherent singlet-triplet interconversion (Fig. 1), driven by the dynamical mixing of the spin eigenstates of coupled free-radicals. The RP spin dynamics are governed by internal magnetic interactions, usually electron-nuclear hyperfine interactions (HFIs) and applied magnetic fields. External static and oscillating magnetic fields can alter RP spin dynamics by Zeeman and HFI resonance effects, and thereby change the relative yields of reaction products that derive, alternatively, from singlet and triplet RP states^18^,^19^. The product yields will depend on the strength of the static field and on the frequency, amplitude, and orientation (relative to the static field) of the applied oscillating magnetic field^17^. These experimental parameters can be adjusted to alter the relative ROS product yields *in vitro*. In this work, we demonstrate magnetic field-induced changes in ROS levels that leave a metabolic signature at the cellular level. We report the effects of the orientation of oscillating fields on the ROS reaction yields in cell cultures and the impact of the resulting variations in the ROS products on the regulation of cellular bioenergetics.

We assume that magnetically sensitive ROS formation occurs at flavoenzyme centers (Fig. 1), initialized by an electron transfer that activates molecular oxygen (O_2_) in reduced flavoenyzmes^20^. A triplet-born, spin-correlated RP is created between flavin semiquinone (FADH^•^) and superoxide (O_2_^•−^)^21^,^22^. The FADH^•^:O_2_^•−^ intermediate is a branch point for ROS products, where subsequent spin-selective reactions have two competing pathways. Either O_2_^•−^ is released from the triplet RP state and escapes into the medium, or a hydroperoxy-flavin transient is formed from the singlet RP state. A subsequent second electron transfer releases hydrogen peroxide (H_2_O_2_). Besides kinetic requirements^22^, the coherent evolution between the singlet and triplet states of FADH^•^:O_2_^•−^ RP determines the amount of the products for the two reaction channels. We hypothesize that RP spin dynamics can be altered by external static and oscillating magnetic fields^23^, which change singlet-triplet intersystem crossing (ISC) rates, and thus affect the outcome of cellular ROS product ratios.

To test the RPM hypothesis for ROS-related quantum biology, we developed low-frequency, low-field instrumentation for product-yield detected magnetic resonance (PYDMR)^21^. In the present PYDMR study, primary human umbilical vein endothelial cells (HUVECs) were exposed to either 50 μT static magnetic fields (SMF) in control samples, or to 50 μT SMFs combined with 1.4 MHz, 20 μT_rms_ RF magnetic fields at Zeeman resonance in experimental samples. The RF orientation was applied in parallel or perpendicular angles with respect to the SMF. Intracellular O_2_^•−^ and extracellular H_2_O_2_ were measured by selective assays in HUVEC cultures. Figure 2 exhibits the changes observed in the ROS product distributions measured for parallel and perpendicular RF orientations. In the perpendicular orientation, we measured a 36% ± 4 reduction of O_2_^•−^ (p < 0.002) and a 21% ± 5 reduction of H_2_O_2_ (p < 0.02). For the parallel orientation, we measured a 10% ± 2 (p < 0.002) reduction of O_2_^•−^ and an 8% ± 8 increase oaf H_2_O_2_ (p < 0.02). Changes in ROS products in cell cultures between SMF ± RF are indicative of a resonance effect on singlet-triplet intersystem crossing (or quantum coherences) at the point of ROS formation^21^.

Singlet-triplet product yields were modeled by numerical simulations of a triplet-born one-proton radical-pair reaction with static and RF magnetic fields at Zeeman resonance^7^, Fig. 3. The numerical results predict an orientation-dependent increase in singlet product yield (H_2_O_2_) and decrease in triplet product yield (O_2_^•−^) when the resonant RF field is added to the static field. These simulations are in agreement with perpendicular experimental results (Fig. 2) and are consistent with our previous cellular results with hyperfine resonance effects^21^. The theoretical model of a one-proton RP does not account for changes in ROS yields (O_2_^•−^ and H_2_O_2_) in the parallel orientation, which suggests either limitations of the simplified model in the complex cellular environment or anisotropic hyperfine resonances near the Zeeman resonance.

To understand the potential effects of ROS signaling on cellular bioenergetics, we measured the oxygen consumption rate (OCR) and extracellular acidification rate (ECAR) with a Seahorse XF Analyzer, Fig. 4. OCR indicates the mitochondrial respiration rate and ECAR is predominately a measure of lactic acid formed during glycolytic energy metabolism^24^,^25^. Figure 4 illustrates experiments in HUVECs that show RF orientation dependence of OCR and ECAR bioenergetic measurements, which appear to result from changes in ROS partitioning. Zeeman resonance at perpendicular excitation did not affect OCR levels (Fig. 4A) but increased ECAR by 100% ± 13 (Fig. 4C). Zeeman resonance at parallel excitation had an opposite effect, where OCR increased by 54% ± 4 (Fig. 4B) while ECAR remained unchanged (Fig. 4D). Altered OCR and ECAR values indicate phenotypic changes in HUVECs bioenergetic pathways, demonstrating spin biochemistry intervention of ROS signaling by RF magnetic fields at Zeeman resonance.

The orientation effects that lead to specific ROS product distributions are in agreement with the simple RPM model for the perpendicular orientation, which predicts an increase in singlet yields with a concomitant decrease in triplet yields, Fig. 3. The overall reduction in measured ROS products at perpendicular excitation suggests a possible ROS signaling link to increased glycolytic activity^26^,^27^. The decrease in steady-state ROS levels with enhanced glycolytic activity might be due to scavenging of ROS by pyruvate^28^. The differential effect on ROS product yields at parallel excitation indicate a signaling channel of increased mitochondrial respiration. Our experiments did not explore the downstream targets of the ROS signaling pathways that lead to regulation of glycolysis and respiration. We suggest that ROS signaling channels are likely to involve the activation of bioenergetic regulation proteins^26^,^29^,^30^, eliciting a type of hormesis response^31^ or a Warburg effect^27^.

Magnetic stimulation of ROS partitioning in live cells reveals hallmarks of quantum coherence, manifested by changes in cellular ROS product distributions. Changes in ROS product partitioning, in turn, altered cellular bioenergetics by increasing either mitochondrial respiration or glycolysis. ROS formation is inextricably linked to bioenergetics by normal cellular metabolism, connecting persistent quantum effects in ROS oxidative signaling to cellular bioenergetics. Because of the importance of ROS in human health and disease^32^, this research is likely to have significance for several areas in bio-medical research. Our results provide fundamental insights into the role of the RPM in ROS redox biology and cellular bioenergetics, revealing a new example of quantum biology.

## Methods

### Cell Culture

Human umbilical vein endothelial cells (HUVECs) (passage 2–4) were cultured in endothelial growth medium (Promocell GmbH, Heidelberg, Germany) supplemented with 2% fetal calf serum (FCS), 0.004 ml/ml endothelial cell growth supplement/heparin, 0.1 ng/ml epidermal growth factor (EGF), 1 ng/ml basic fibroblast growth factor, and 1 mg/ml hydrocortisone at 37 °C with 5% CO_2_. The cells were cultured in a 75-cm^2^ flask to expand cell number. After reaching confluence, the cells were seeded in (a) 6-well culture plates at a density of 3.5×10^3^ cells/cm^2^ for ROS experiments; (b) and in mini plates at 3,500 cells per well for Seahorse XFp Analyzer (Agilent Technologies, Santa Clara, CA).

### ROS Measurements

Cellular H_2_O_2_ production was measured with the horseradish peroxidase-linked Amplex Ultra Red (HRP-AUR) fluorometric assay (Invitrogen). After cells were seeded, they were exposed to SMF ± RF magnetic fields for the duration of the experiment, by use of the PYDMR apparatus we previously described^21^. At day 3, medium was aspirated and cells were washed with Hank’s Buffer (HB) with 100 μM diethylenetriaminepentaacetic acid (DTPA) and incubated for 1 h with HB containing 10 μM AUR and 0.2 units/ml HRP. Resorufin fluorescence was collected on a Gemini fluorescence microplate reader (Molecular Devices, Sunnyvale, CA).

To measure intracellular production of O_2_^•−^, HUVECs were incubated for 30 min at 37 °C in HB containing 50 μM dihydroethidium (DHE) at day 3. Cells were washed twice in HB with 100 μM DTPA and harvested in 300 μL of methanol. The samples were spun down for 15 min at 12,000 rpm at 4 °C, the supernatant was collected and dried for 5–6 hrs at 37 °C and stored at −20 °C for 7–10 days before high-performance liquid chromatography (HPLC) analysis. An Agilent 1100 series HPLC with a G1321A fluorescence detector was used for DHE product separation. Analytes were separated using a C-18 reverse-phase column (Nucleosil 250 to 4.5 mm) as previously described^33^. The total fluorescence intensity of the 2-hydroxyethidium peak were averaged for all wells and then averaged for three separate experiments.

Protein content, HPLC analysis, and resorufin fluorescence were measured at the same termination points. H_2_O_2_ and O_2_^•−^ measurements were normalized to total protein concentration (BCA, Pierce). H_2_O_2_ calibration curves with HRP-AUR under the same RF magnetic field strengths showed no differences compared to SMF controls, demonstrating that RF fields do not interact with the H_2_O_2_ detection assay.

### Cellular Bioenergetics

An XFp Analyzer from Seahorse Bioscience was used for the bioenergetic measurements. HUVECs were seeded at 4,000 cells per well in XFp miniplates and exposed to RF magnetic fields for 2 days. The MitoStress Test Assay was performed as described previously^24^,^25^. The XFp Analyzer measured mitochondrial respiration and glycolysis *in vitro*^34^,^35^ in the same samples. Mitochondrial respiration was determined by the oxygen consumption rate (OCR) in cell culture. Glycolysis was assessed by the extracellular acidification rate (ECAR). To estimate the proportion of the OCR coupled to ATP synthesis, oligomycin (1 μg/ml) was injected at 21 minutes into all samples to inhibit the ATP synthase. Typically, the OCR rate decreases in response to oligomycin by the extent to which the cells are using mitochondria to generate ATP. The remaining OCR can be ascribed to both proton leak across the mitochondrial membrane and oxygen consumption by processes other than reduction at cytochrome c oxidase. To determine the maximal OCR that the cells can sustain, the proton ionophore (uncoupler) FCCP (1 μM) was injected at 41 minutes. Lastly, antimycin A (10 μM) is injected at 61 minutes to inhibit electron flux through Complex III, which suppresses OCR dramatically. What remains is the OCR that is attributable to O_2_ consumption due to the formation of mitochondrial ROS and non-mitochondrial sources.

## Additional Information

**How to cite this article**: Usselman, R. J. *et al*. The Quantum Biology of Reactive Oxygen Species Partitioning Impacts Cellular Bioenergetics. *Sci. Rep.* **6**, 38543; doi: 10.1038/srep38543 (2016).

**Publisher's note:** Springer Nature remains neutral with regard to jurisdictional claims in published maps and institutional affiliations.

## Figures and Tables

**Figure 1:**
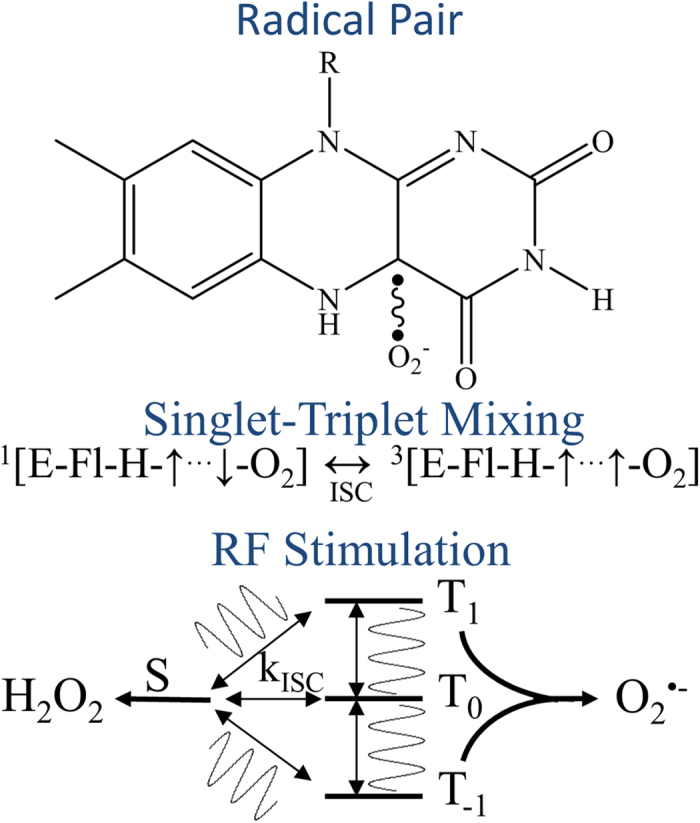
Flavin semiquinone (FADH^•^) and superoxide (O_2_^•−^) spin-correlated radical pair (top). The radical pair undergoes intersystem crossing (ISC) between singlet (S) and triplet states (T_-1,0,1_) at rates (k_ISC_) determined by electron-nuclear hyperfine and Zeeman interactions (middle). An applied RF oscillating magnetic field tuned at Zeeman resonance (1.4 MHz at 50 μT magnetic field), modifies k_ISC_ (bottom), and ultimately affects the relative amount of singlet (H_2_O_2_) and triplet (O_2_^•−^) reaction products. Resonance effects on ROS product yields are a key manifestation of quantum biology.

**Figure 2:**
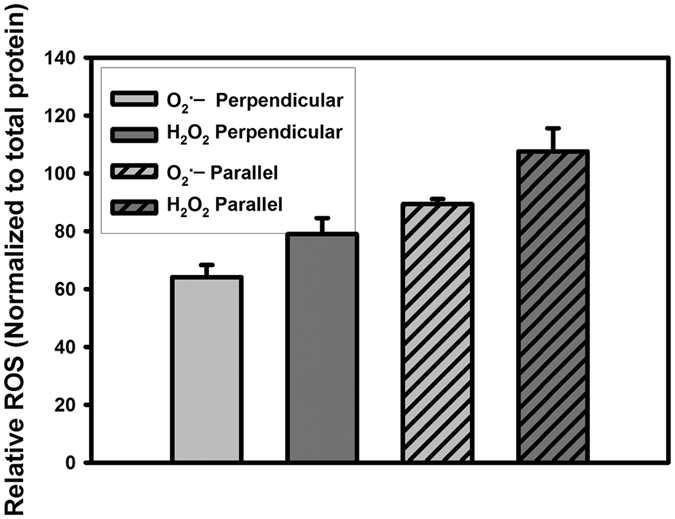
ROS product distributions illustrating the RF magnetic field orientation dependence at Zeeman resonance (1.4 MHz and 50 μT magnetic field). The data is presented as a product yield ratio percentage with ± RF in the static field. RF magnetic fields in perpendicular orientation decreased both O_2_^•−^ and H_2_O_2_ levels by 36% ± 4% (p < 0.002) and 21% ± 5% (p < 0.02), respectively, relative to controls. In parallel orientation, H_2_O_2_ levels increased by 8% ± 8% (p < 0.02), whereas O_2_^•−^ decreased by 10% ± 2% (p < 0.002), relative to controls. The amount of ROS produced were normalized to protein concentrations and represent the average of 3 independent experiments.

**Figure 3:**
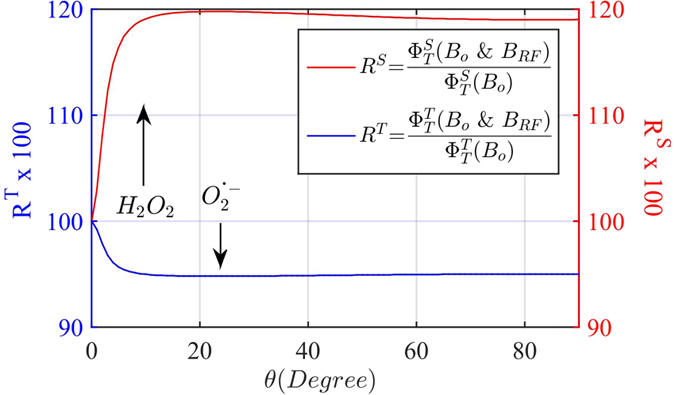
Numerical simulations of the effects of RF magnetic fields at Zeeman resonance on triplet (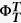, O_2_^•−^) and singlet (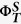, H_2_O_2_) yields of a triplet-born radical-pair reaction. The RP model shown here is for a one-proton radical-pair with one isotropic hyperfine coupling of 500 μT and a RP lifetime of 10 μs. The static magnetic field was set to B_o_ = 50 μT and the RF magnetic field amplitude set to B_RF_ = 20 μT (RMS), with a Zeeman resonance frequency of 1.4 MHz. R^T^ (R^S^) is the relative change of triplet (singlet) yields produced by the RF, relative to the levels in the static field.

**Figure 4:**
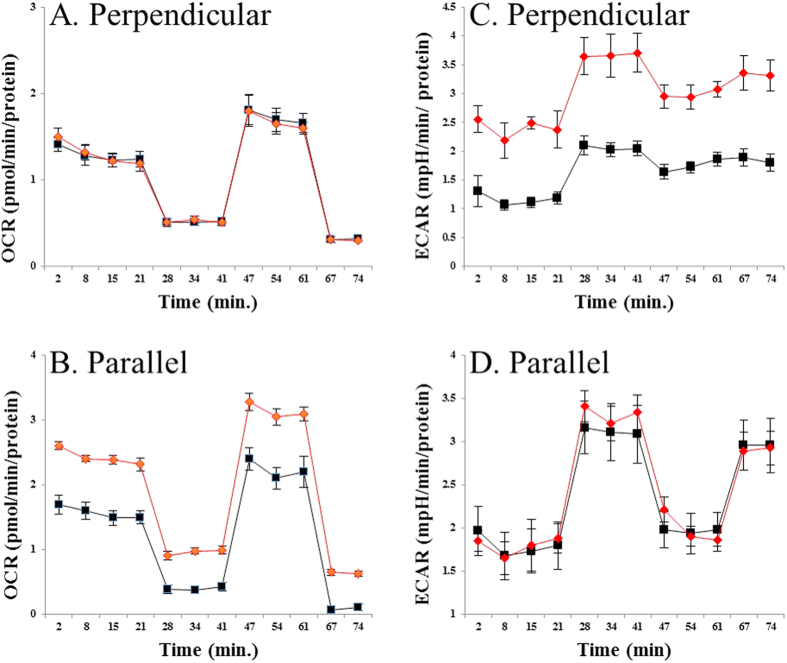
Bioenergetic profiles of HUVECs cultures monitored with a Seahorse XF Analyzer, comparing static alone (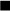) and static + RF (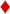) magnetic fields. Oligomycin was added at 21 min to inhibit ATP synthesis, the proton ionophore FCCP was added at 41 min to assess the maximum possible oxygen consumption, and antimycin A was added at 61 min to inhibit electron flow through complex III. The static magnetic field was 50 μT and the RF was 1.4 MHz at Zeeman resonance. The RF was oriented perpendicular (**A** and **C**) or parallel (**B** and **D**) to the static field. Perpendicular RF excitation did not affect the oxygen consumption rate (OCR) (Panel A), whereas extracellular acidification rate (ECAR) was increased by 100 ± 13 (p < 0.001, Panel C). Parallel RF excitation lead to increased OCR by 54 ± 4 (p < 0.001, Panel B), while ECAR remained unchanged (Panel D). These are results of 3 independent representative experiments and data were normalized to total cellular protein.
